# Trending ability of electrical cardiometry for non-invasive cardiac output monitoring in preterm neonates during the transitional period: a polar plot analysis

**DOI:** 10.1007/s00431-026-07117-9

**Published:** 2026-06-04

**Authors:** Silvia Martini, Mariarosaria Annunziata, Jacopo Lenzi, Emma Coppi, Lizelle van Wyk, Luigi Corvaglia

**Affiliations:** 1 Neonatal Intensive Care Unit, IRCCS S. Orsola-Malpighi Hospital, Bologna, Italy; 2 Department of Medical and Surgical Sciences, University of Bologna, Bologna, Italy; 3 Department of Biomedical and Neuromotor Sciences, University of Bologna, Bologna, Italy; 4 Department of Paediatrics & Child Health, Stellenbosch University, Cape Town, South Africa

**Keywords:** Electrical cardiometry, Cardiac output, Transthoracic echocardiography, Trending analysis, Preterm infants

## Abstract

Electrical cardiometry (EC) enables continuous, non-invasive monitoring of cardiac output (CO) in neonates. The ability of this technique to track CO changes over time compared to transthoracic echocardiography (TTE) remains poorly studied in the neonatal population. Using polar plot analysis, we aimed to assess the trending ability of EC compared with TTE in preterm neonates during the transitional period. CO measurements were performed on preterm infants < 32 weeks’ gestation and/or < 1500 g on day 1, 2 and 3 of life. Paired changes in CO (ΔCO) between consecutive days of life were calculated for both EC and TTE and used for polar plot analysis. Overall, 55 paired ΔCO measurements were available for day 1–2 and 45 for day 2–3. After excluding changes < 20% of the mean absolute ΔCO value, 46 and 41 pairs were included in the polar analysis. EC demonstrated good trending performance, with 87% and 88% of points falling within ± 30° for day 1–2 and day 2–3, respectively. The mean angular bias was 0.6° (95% CI − 5.7° to 6.8°) for days 1–2 and − 7.2° (95% CI − 16.4° to 2.0°) for days 2–3, indicating minimal systematic directional disagreement between methods.

*Conclusion*: Our findings suggest a good trend tracking performance of EC for non-invasive CO monitoring during the transitional period in preterm neonates, supporting its potential role as a continuous monitoring tool for neonatal hemodynamic assessment.

**Table:** 

**What Is Known:** *• Electrical cardiometry (EC) allows continuous, non-invasive cardiac output (CO) monitoring in neonates. * *• While EC accuracy for CO assessment has been previously investigated, little is known on its trending ability. * **What Is New:** *• EC showed good ability to track CO changes in preterm neonates when compared to transthoracic echocardiography.* *• Over 85% of paired ΔCO were within ±30°, with minimal angular bias. *

**What Is Known:**

*• Electrical cardiometry (EC) allows continuous, non-invasive cardiac output (CO) monitoring in neonates.
*

*• While EC accuracy for CO assessment has been previously investigated, little is known on its trending ability.
*

**What Is New:**

*• EC showed good ability to track CO changes in preterm neonates when compared to transthoracic echocardiography.*

*• Over 85% of paired ΔCO were within ±30°, with minimal angular bias.
*

## Introduction

Accurate cardiac output (CO) assessment is crucial for the hemodynamic management of preterm infants, as early detection of reduced systemic perfusion may prevent severe complications and reduce neonatal mortality. Transthoracic echocardiography (TTE) is the current non-invasive gold standard for CO assessment; however, its intermittent nature and operator dependency represent important limitations. Consequently, non-invasive CO monitoring techniques have gained increasing interest in neonatal care [1].

Electrical cardiometry (EC) is a transthoracic bioimpedance-based technology that continuously estimates CO by analysing pulsatile impedance changes associated with peak aortic blood flow acceleration. Neonatal validation studies comparing EC and TTE have produced inconsistent findings. Although the mean bias is generally small, wide limits of agreement are common, with percentage errors often exceeding accepted thresholds for clinical interchangeability [2]. The presence of intra- and extra-cardiac shunts, common during the first 72 h after birth, may further compromise EC accuracy for absolute CO estimation [3–5]. Despite limited interchangeability with TTE for absolute measurements, EC may still provide clinically relevant information on directional CO changes over time.

This study aimed to evaluate, using polar plot analysis, the trending ability of EC compared with TTE in very preterm infants during the transitional period.

## Methods

This study is a sub-analysis of the prospective NEO-ICM project, enrolling preterm infants < 32 weeks’ gestation and/or < 1500 g admitted to the Neonatal Intensive Care Unit of IRCCS AOUBO (Bologna, Italy). Major congenital malformations, congenital heart disease, or clinical conditions potentially affecting study parameters (i.e. transfusion-requiring anaemia and persistent pulmonary hypertension requiring inhaled nitric oxide) were exclusion criteria. The study was approved by the Ethics Committee of S. Orsola-Malpighi Hospital (328/2017/O/Oss) and conducted in accordance with the Declaration of Helsinki. Written informed parental consent was obtained.

EC monitoring of cardiac output (CO_EC_) was performed over the first 72 h of life using the ICON® device (Osypka Medical Inc., Berlin, Germany) with beat-to-beat sampling. Neonatal sensors were applied according to manufacturer instructions. Recordings were reviewed for artefacts and signal quality; optimal signals were defined by a signal quality index > 80 [6]. CO_EC_ values were averaged over the 30-s interval preceding TTE to avoid signal interference.

CO_TTE_ measurements were performed daily by a single experienced operator, blinded to EC data, using a CX50 ultrasound scanner (Philips Healthcare, Amsterdam, The Netherlands) with a 12-MHz probe. CO_TTE_ was calculated as: (left ventricular outflow [LVO] × velocity–time integral [VTI]) × heart rate × LVO cross-sectional area. LVO tract diameter was usually measured from the parasternal long-axis view with the leading-edge method, at the hinge points of the aortic valve at end-systole on 2D images, as per current recommendations [7]. VTI was obtained from an apical five-chamber view using pulsed-wave Doppler, with angle correction when required. For each examination, CO_TTE_ values were averaged over five cardiac cycles. Ductal status was classified as haemodynamically significant (hsPDA) in the presence of a pulsatile shunt pattern (end-diastolic to peak-systolic velocity ratio ≥ 0.5) and left-atrium-to-aortic-root (LA:Ao) ratio ≥ 1.5 and/or evidence of reversed end-diastolic flow in the abdominal aorta and/or in the anterior cerebral artery.

Both CO_EC_ and CO_TTE_ were indexed to body weight (ml/kg/min). Relevant clinical variables potentially influencing CO estimation were also recorded.

For each infant, CO changes between days 1–2 and 2–3 were calculated separately for EC (ΔCO_EC_) and TTE (ΔCO_TTE_) and used for statistical analysis. To avoid cumulative physiological effects unrelated to short-term trending, changes between days 1–3 were not analysed.

### Statistical analysis

Trending ability was evaluated using polar plot analysis according to Critchley et al. [8, 9], which enables assessment of both the direction and magnitude of ΔCO changes between measurement methods. Each paired change was represented as a vector from the origin, with radius *r* = (|ΔCO_EC_| +|ΔCO_TTE_|)/2 and angle *θ* = arctan[(ΔCO_EC_ – ΔCO_TTE_)/r]. Vectors were rotated 45° clockwise so the line of identity coincided with the horizontal axis. Angles were expressed symmetrically around zero. Vectors with a radial magnitude (mean ΔCO) below 20% of the overall mean ΔCO were excluded to reduce noise from minimal changes. This threshold was applied to the polar radius, not to absolute CO values, and was selected as a pragmatic central exclusion zone, consistent with the rationale proposed by Critchley et al. [8, 9], according to which near-origin data should be excluded because very small ΔCO values are dominated by random error.

Data were displayed on polar coordinates, where radial distance indicates magnitude and angular deviation reflects directional agreement. Following Critchley et al. [8, 9], angular limits of acceptable trending agreement were set a priori at ± 30°. These limits refer to angular disagreement between paired ΔCO vectors, not to the clinical acceptability of absolute CO changes.

Concordance rates were calculated as the proportion of points within ± 30°, with 95% confidence intervals estimated using the Agresti–Coull method. Polar plots were generated using Stata 18 (StataCorp, College Station, TX, USA). Angular bias (mean *θ*) and radial dispersion were visually assessed to evaluate the ability of EC to track directional CO changes.

## Results

Fifty-five preterm infants, enrolled between 2018 and 2021, provided 55 paired ΔCO measurements for days 1–2 and 45 for days 2–3. Clinical characteristics of the study population are reported in Table 1.

**Table 1:** Clinical characteristics of the study population

Baseline characteristics (*n* = 55)			
Gestational age (weeks), mean (SD)	29.3 (2.6)		
Birth weight (g), mean (SD)	1175 (357)		
Sex (males), *n* (%)	28 (51)		
Small for gestational age, *n* (%)	11 (20)		
Type of delivery (C-section), *n* (%)	48 (87)		
CRIB-II score, mean (SD)	8 (4)		
Apgar score at 5 min, mean (SD)	8.6 (1.3)		
Monitoring period (days of life)	**Day 1** (*n* = 55)	**Day 2** (*n* = 55)	**Day 3** (*n* = 45)
CO_EC_ (ml/kg/day), mean (SD)	286 (81)	290 (66)	274 (72)
CO_TTE_ (ml/kg/day), mean (SD)	297 (82)	299 (63)	287 (71)
Cardiac shunts, *n* (%)			
Hemodynamically significant PDA	30 (55)	15 (27)	7 (16)
Patent foramen ovale	53 (96.4)	53 (96.4)	44 (97.8)
Ongoing cardiovascular drugs, *n* (%)			
Dobutamine	11 (20)	13 (24)	9 (20)
Dopamine	8 (15)	7 (13)	4 (9)
Surfactant administration, *n* (%)	33 (60)	35 (64)	35 (78)
Respiratory support, *n* (%)			
Invasive ventilation	14 (25)	13 (24)	9 (20)
nCPAP or Bilevel	38 (69)	35 (64)	25 (56)
Nasal cannulas or self-ventilating in air	3 (5)	7 (13)	11 (24)
Haemoglobin (g/dl), mean (SD)	15.5 (2.3)	15.1 (2.7)	14.9 (2.9)

*SD* standard deviation, *CRIB-II* Clinical risk index for babies II, *CO*_*EC*_ cardiac output monitored with electrical cardiometry, *CO*_*TTE*_ cardiac output monitored with transthoracic echocardiography, *nCPAP* nasal continuous positive airway pressure, *PDA* patent ductus arteriosus

Between days 1–2, ΔCO_TTE_ ranged from − 159 to + 146 ml/kg/min (mean ± SD: 6.3 ± 68.1), while ΔCO_EC_ ranged from − 178 to + 117 ml/kg/min (4.1 ± 68.0). Between days 2–3, ΔCO_TTE_ ranged from − 199 to + 147 ml/kg/min (− 15.3 ± 76.4) and ΔCO_EC_ from − 188 to + 154 ml/kg/min (− 12.5 ± 70.1).

**Fig. 1:**
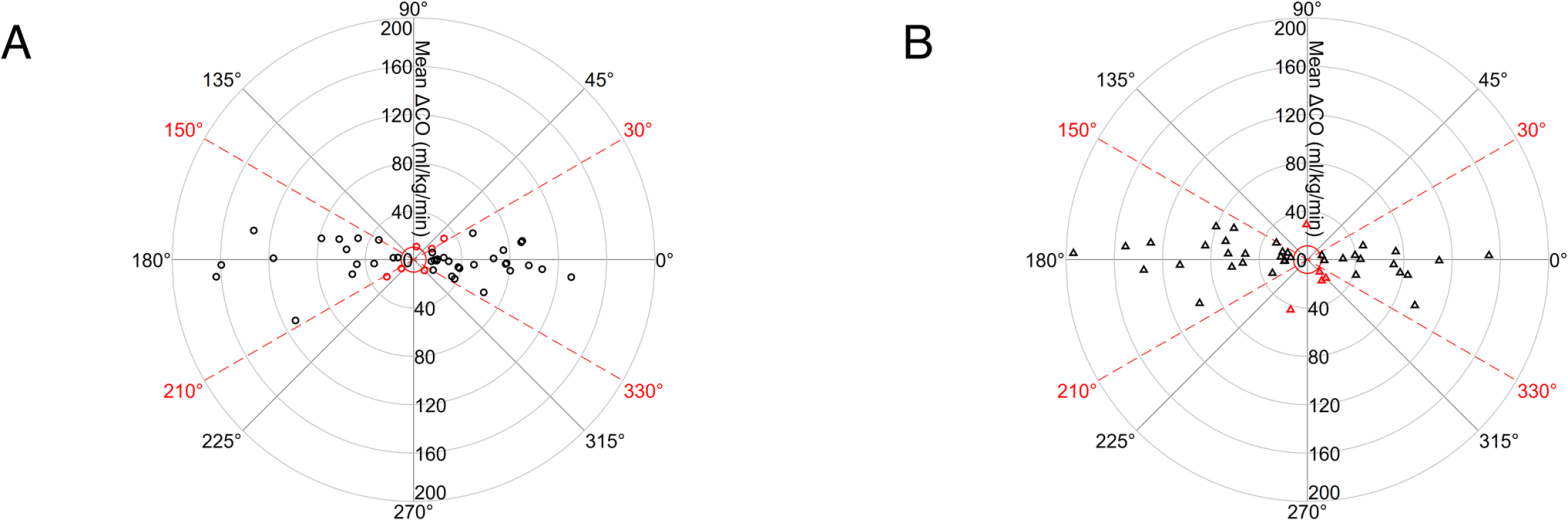
Polar plot of cardiac output changes (ΔCO), measured by electrical cardiometry and transthoracic echocardiography, between days 1–2 (**A**) and days 2–3 (**B**). Red markers indicate points outside the ± 30° limits of agreement; the dashed lines represent the ± 30° limits. The red circle denotes the 20% exclusion zone around the mean ΔCO

The mean absolute ΔCO was 51.7 ± 41.6 ml/kg/min for days 1–2 and 57.0 ± 45.5 ml/kg/min for days 2–3. Changes with a mean ΔCO < 20% of the overall mean ΔCO (10.3 and 11.4 ml/kg/min, respectively) were excluded to minimise noise, leaving 46 and 41 paired observations for polar plot analysis, illustrated in Fig. 1.

For days 1–2, 40/46 data points (87%) fell within the ± 30° limits of agreement and 6 (13%) outside. For days 2–3, 36/41 points (88%) were within the limits and 5 (12%) outside. Concordance rates were 87% (95% CI 74–94%) and 88% (95% CI 74–95%), respectively. Angular deviations were symmetrically distributed around 0°, forming a typical butterfly pattern. Angular bias was minimal: 0.6° (95% CI − 5.7° to 6.8°) for days 1–2 and − 7.2° (95% CI − 16.4° to 2.0°) for days 2–3, indicating no significant systematic directional disagreement.

Outliers showed no clustering by patent ductus arteriosus status or inotropic therapy for either time interval; however, given the small number of outliers, no formal statistical comparisons were performed.

## Discussion

This study evaluated the trending ability of EC compared with TTE using polar plot analysis and demonstrated good capacity to track directional CO changes in preterm neonates during the transitional period. Across both intervals, more than 85% of observations fell within the ± 30° limits of agreement, with minimal angular bias and a symmetrical distribution around 0°. These findings indicate that EC can reliably follow both the direction and approximate magnitude of CO variations, supporting its potential role in the hemodynamic monitoring of preterm infants.

Our results help address an important gap in neonatal hemodynamic monitoring. While most previous neonatal validation studies of electrical biosensing technologies have focused on point-estimate agreement with TTE using Bland–Altman analysis or correlation metrics, evidence on trending ability remains scarce [2]: to date, only one study assessed trending performance using bioreactance, reporting poor concordance and substantial angular bias [10]. Several methodological factors may explain the discrepancy with our results. First, bioreactance and EC rely on different bioimpedance principles, potentially affecting responsiveness to rapid hemodynamic changes. Second, the use of a central exclusion zone, consistent with the rationale of polar plot methodology [8], likely improved reliability by reducing noise from very small CO fluctuations. Third, our study examined day-to-day changes, whereas previous work analysed higher-frequency repeated measurements, potentially increasing variability and reducing apparent concordance.

Our results align with paediatric data from children undergoing cardiac surgery, in whom EC demonstrated good trending ability despite limited interchangeability for absolute CO values [11]. Together with our companion agreement analysis from the same cohort showing a mean percentage error well below the 30% threshold [5], these findings provide complementary evidence supporting both accuracy and trending performance, key prerequisites for clinical implementation of EC. The use of a single experienced operator for TTE measurements may have further reduced inter-operator variability and contributed to the observed performance.

Although TTE is the reference method for non-invasive neonatal CO assessment, it is not an ideal gold standard due to intra- and inter-operator variability [12]. More accurate techniques such as transpulmonary thermodilution are generally unsuitable for preterm infants because of their invasiveness. In exploratory analyses, outliers outside the ± 30° limits did not cluster according to patent ductus arteriosus status or inotropic therapy, suggesting no clear association with these factors; however, the small number of outliers precluded formal statistical testing, therefore these observations should be interpreted with caution.

This study has limitations. The relatively small, single-centre cohort may limit generalizability. Trending analysis was based on daily measurements, capturing transitional changes but not rapid hemodynamic fluctuations, which may represent the clinical setting where continuous monitoring is most valuable. Accordingly, the ± 30° threshold should be interpreted as a methodological benchmark for trending performance rather than as a neonatal clinical threshold; whether EC can reliably detect clinically relevant low flow states requires targeted evaluation in cohorts enriched for such events. Analyses of outliers were descriptive only, and the 20% exclusion zone, while consistent with the rationale of polar plot methodology, represents a pragmatic and relatively conservative choice.

In conclusion, this study provides the first neonatal evidence, using formal polar plot methodology, that EC can reliably track directional CO changes during postnatal transition. These findings support EC as a trend-monitoring tool within multimodal neonatal hemodynamic monitoring, particularly when TTE is not continuously available. Future research should evaluate EC performance during rapid hemodynamic changes and in more complex clinical scenarios.

## Data Availability

Data are available from the corresponding author upon reasonable request.

## Abbreviations

CI: Confidence interval
CO: Cardiac output
CO_TTE_: Cardiac output measured by echocardiography
CO_EC_: Cardiac output measured by electrical cardiometry
EC: Electrical cardiometry
hsPDA: Hemodynamically significant patent ductus arteriosus
LA:AO ratio: Left-atrium-to-aortic-root ratio
LVO: Left ventricular outflow
TTE: Transthoracic echocardiography
VTI: Velocity time integral

## Code availability

N/A.
